# Contemporary Neoadjuvant Therapies for High-Risk Melanoma: A Systematic Review

**DOI:** 10.3390/cancers13081905

**Published:** 2021-04-15

**Authors:** Kerianne Boulva, Sameer Apte, Ashley Yu, Alexandre Tran, Risa Shorr, Xinni Song, Michael Ong, Carolyn Nessim

**Affiliations:** 1 Division of General Surgery, The Ottawa Hospital, Ottawa, ON K1H 8L6, Canada; kboulva@toh.ca (K.B.); sapte@ohri.ca (S.A.); aletran@toh.on.ca (A.T.); 2 The Ottawa Hospital Research Institute, Ottawa, ON K1H 8L6, Canada; rshorr@toh.ca (R.S.); xsong@toh.ca (X.S.); mong@toh.ca (M.O.); 3 Department of Family Medicine, McMaster University, Hamilton, ON L8P 1H6, Canada; ashley.yu@uottawa.ca; 4 Division of Medical Oncology, The Ottawa Hospital, Ottawa, ON K1H 8L6, Canada

**Keywords:** melanoma, neoadjuvant, targeted therapy, immunotherapy, intralesional therapy

## Abstract

**Simple Summary:**

The risk of relapse for stage III melanoma remains high. Subsequently, there has been a surge of interest in the role of contemporary therapies in the neoadjuvant setting. Results of 8 phase II trials show safety, remarkable pathologic response and relapse-free survival.

**Abstract:**

Despite advances in adjuvant immuno- and targeted therapies, the risk of relapse for stage III melanoma remains high. With 43 active entries on clinicaltrials.gov (8 July 2020), there is a surge of interest in the role of contemporary therapies in the neoadjuvant setting. We conducted a systematic review of trials performed in the last decade evaluating neoadjuvant targeted, immuno- or intralesional therapy for resectable stage III or IV melanoma. Database searches of Medline, Embase, and the Cochrane Central Register of Controlled Trials were conducted from inception to 13 February 2020. Two reviewers assessed titles, abstracts, and full texts. Trials investigating contemporary neoadjuvant therapies in high-risk melanoma were included. Eight phase II trials (4 randomized and 4 single-arm) involving 450 patients reported on neoadjuvant anti-BRAF/MEK targeted therapy (3), anti-PD-1/CTLA-4 immunotherapy (3), and intralesional therapy (2). The safest and most efficacious regimens were dabrafenib/trametinib and combination ipilimumab (1 mg/kg) + nivolumab (3 mg/kg). Pathologic complete response (pCR) and adverse events were comparable. Ipilimumab + nivolumab exhibited longer RFS. Contemporary neoadjuvant therapies are not only safe, but also demonstrate remarkable pCR and RFS—outcomes which are regarded as meaningful surrogates for long-term survival. Studies defining predictors of pCR, its correlation with oncologic outcomes, and phase III trials comparing neoadjuvant therapy to standard of care will be crucial.

## 1. Introduction

With 96,480 new cases per year, melanoma is the 5th leading cause of cancer in the Unites States. Worldwide, its incidence has increased over the past 50 years [1,2]. Although lower-risk melanomas (stage I, IIA-B and IIIA) are highly curable with 10-year overall survival (OS) rates of 82% to 98%, higher risk melanomas (stage IIC and IIIB-D) have worse risk of relapse and 10-yr OS rates of 24% to 77% [3].

In the last decade, randomized controlled trials using anti-BRAF/MEK targeted therapies such as dabrafenib/trametinib (DAB + TRAM) or vemurafenib/cometinib, and anti-PD-1/CLTA-4 checkpoint inhibitor immunotherapy such as ipilimumab (IPI) or nivolumab (NIVO) have demonstrated dramatic improvements in progression-free survival (PFS) and OS for unresectable melanoma [4,5,6]. Traditionally, chemotherapy and interleukin-2 were standards of care for advanced melanoma. Chemotherapy, however, has never demonstrated a survival advantage [7,8,9]. Similarly, interleukin-2 benefits only a small minority of patients and is highly toxic [10,11]. As a result, the advent of immuno- and targeted therapy has revolutionized the treatment paradigm for stage IV melanoma, giving hope and longer life to patients with advanced melanoma.

Encouraged by results for advanced melanoma, subsequent trials evaluated the adjuvant role of immuno- and targeted therapies for completely resected stage III disease. For patients with a BRAF^V600E/K^ mutation, combination DAB + TRAM showed improved relapse-free survival (RFS) and OS when compared to placebo (HR for recurrence or death 0.47, 95%CI [0.39, 0.58]) [12,13]. Immune checkpoint inhibition with IPI also resulted in higher rates of RFS, OS and distant-metastasis free survival (DMFS) when compared to placebo (HR for recurrence or death 0.76, 95%CI [0.64, 0.89]) [14,15]. Similarly, pembrolizumab led to improved RFS and reduced risk of death (HR for recurrence or death 0.57, 98.4%CI [0.43, 0.74]) [16], while NIVO versus IPI showed improved 4-year RFS (HR 0.71, 95%CI [0.60, 0.86] [17]. As a result, clinical practice rapidly changed, defining the current standard of care for lymph node positive resectable stage III disease as upfront surgery followed by adjuvant immuno- or targeted therapy.

Although advances in adjuvant therapy for stage III melanoma are remarkable, the risk of relapse remains high, especially for palpable or radiographically detected nodal disease. Furthermore, neoadjuvant therapy has become standard of care in locally advanced breast, rectal, and esophagogastric cancers [18,19]. The advantages of neoadjuvant therapy are many: (1) reduction of tumor burden and surgical morbidity; (2) potential conversion of unresectable to resectable disease; (3) pathologic and radiological assessment of treatment response; (4) higher treatment completion rates; and (5) collection of specimens for translational research [20,21]. More importantly, T-cell immune checkpoint blockade potentially acts synergistically with other systemic therapies in unresected disease, by inducing a stronger tumor-specific T-cell response [21]. For these reasons, there has been a surge of interest in the role of contemporary therapies in the neoadjuvant setting for melanoma. At present, there are 43 active, planned, or ongoing interventional trials evaluating neoadjuvant approaches in high-risk melanoma registered on clinicaltrials.gov [22].

Contemporary neoadjuvant therapies are poised to change the standard of care for resectable high-risk palpable Stage III melanoma. To characterize progress in the neoadjuvant approach, we conducted a systematic review of phase II and III trials performed in the last decade evaluating neoadjuvant immuno-, targeted-, and intralesional therapy for palpable stage III or oligometastatic stage IV melanoma.

## 2. Materials and Methods

### 2.1. Data Sources

This systematic review was designed according to the PRISMA-P statement. It was reported and conducted as per the PRISMA-P statement and *Cochrane Handbook for Systematic Reviews of Interventions* [23,24]. Database searches were conducted using Medline, Embase, and the Cochrane Central Register of Controlled Trials from inception to 13 February 2020. The complete search strategy can be found in Appendix A. Abstracts from the 2020 *American Society of Clinical Oncology* (ASCO) conference were also reviewed on 31 May 2020. Search terms included: melanoma, neoadjuvant, preoperative, immunotherapy, targeted therapy, talimogene laherparepvec.

### 2.2. Study Selection and Review Process

Eligible studies were included if they met the following criteria: (1) randomized controlled trial or single-arm trial evaluating targeted therapy, immunotherapy or intralesional therapy; (2) conference abstracts consistent with inclusion criteria 1 without an associated manuscript; (3) articles published between 1 January 2009 to February 13 2020, including eligible abstracts from ASCO 2020; (4) and English language publications. We excluded (1) duplicate publications; (2) phase I trials; (3) case reports and series; (4) retrospective studies; (5) animal and ex vivo studies; (6) studies evaluating chemotherapy or biochemotherapy.

Titles and abstracts of all retrieved studies were screened by two independent reviewers (KB, SA) according to the pre-determined inclusion and exclusion criteria. References listed from relevant articles were also screened for additional titles. Disagreement was resolved by discussion and final consensus. Full-text screening was conducted by two reviewers (KB, SA) and reasons for exclusion were recorded. When multiple publications from the same study were available, the most recent results with the largest number of patients was included, unless different data sets or different outcomes were reported.

### 2.3. Data Extraction

Data extraction was systematically performed to produce a descriptive summary of study participants, interventions and outcomes (Table 1). A pre-specified data extraction form was used. KB extracted the data independently, and data integrity was reviewed by SA. Outcomes of interest included clinical or pathologic response, recurrence and survival.

### 2.4. Study Quality Appraisal

Risk of bias for RCTs was assessed using the Cochrane Risk of Bias Tool (Appendix B) [25]. For single-arm trials, a modified version of the Newcastle-Ottawa scale was used (selection and outcome categories) to assess risk of bias in terms of sample selection and outcome assessment (Appendix C) [26]. No authors were contacted for additional study information.

### 2.5. Data Analysis

We collected descriptive statistics for each included study. Due to the variability of the treatment arms, of the reported outcomes, and the paucity of homogeneous randomized data, a meta-analysis was not possible.

## 3. Results

**Figure 1:**
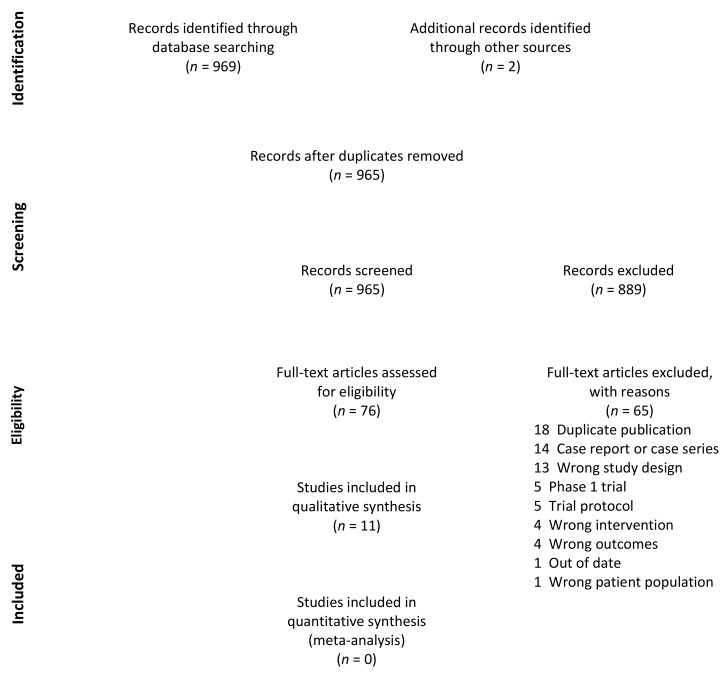
PRISMA flow diagram.

A total of 971 records were identified. Six duplicates were removed. Title and abstract screening was performed on 965 records, 889 of which were deemed irrelevant by the two reviewers. Full-text screening was performed on 76 studies, and a total of 11 references were included for review [20,27,28,29,30,31,32,33,34,35,36]. A PRISMA flow diagram is presented in Figure 1 [24]. Pooling of data in a meta-analysis or quantitative analysis was deemed inappropriate due to the lack of comparative trials, high proportion of single-arm trials, heterogeneity of interventions, discrepancy in outcome definitions, and non-standardized reporting.

**Figure 2:**
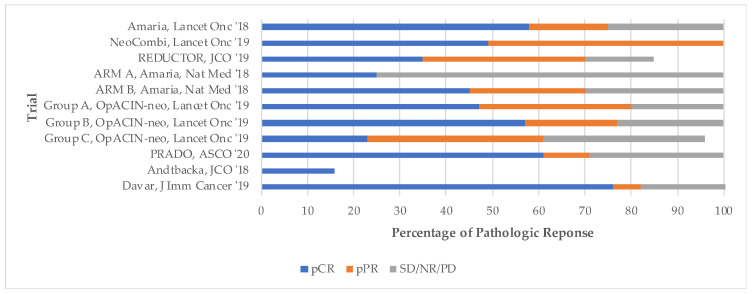
Pathologic response by trial.

**Figure 3:**
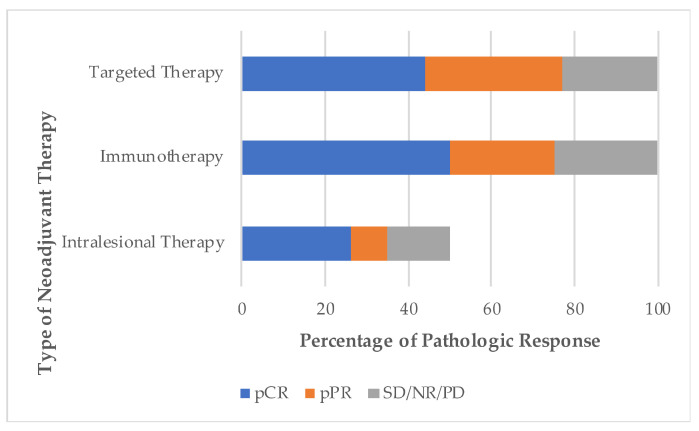
Pathologic response by type of neoadjuvant therapy. pCR, pathologic complete response, no evidence of viable tumor cells on complete pathological evaluation of the surgical specimen. pPR, pathologic partial response, less than 50% viable tumor cells on complete pathological evaluation of the surgical specimen. NR, non response, more than 50% viable tumor in the surgical specimen. SD, stable disease. PD, progression of disease.

Four full-text publications and seven conference abstracts were included, comprising a total of 450 patients across eight trials. Two trials contained data from more than one reference. All studies were phase II trials, four of which were randomized and four of which were single-arm. Trials evaluated neoadjuvant anti-BRAF/MEK targeted therapy (3), anti-PD-1/CTLA-4 checkpoint inhibitor immunotherapy (3), and intralesional therapy (2). Results of these trials are summarized in Table 1. Pathologic response by trial as well as by treatment modality are described in Figure 2 and Figure 3, respectively.

### 3.1. Neoadjuvant Targeted Therapy

Neoadjuvant combination targeted therapy with DAB + TRAM was evaluated in 3 studies. Amaria et al. were the first to compare neoadjuvant plus adjuvant DAB + TRAM plus surgery (treatment group; 14 patients) versus upfront surgery plus consideration of standard of care adjuvant therapy (standard of care group; seven patients) [28]. Neoadjuvant plus adjuvant DAB + TRAM significantly improved DFS when compared to standard of care (upfront surgery and consideration of adjuvant therapy) in patients with resectable stage III-IV BRAF^V600E/K^ mutant melanoma (19.7 months vs. 2.9 months; HR 0.016, *p* < 0.0001). Seven of the 12 patients (58%) who underwent surgery in the treatment group had a pathological complete response (pCR), with a longer DMFS than those without pCR. Neoadjuvant plus adjuvant DAB + TRAM was well tolerated, with only 7% grade 3 adverse events (AEs), no grade 4 AEs, and no treatment-related deaths. Finally, the molecular and immune profiling performed in the treatment group showed tumors achieving pCR had lower baseline pERK positivity, less expression of *TIM-3* and *LAG-3* on CD8+ PD1 T cells, little to no remodelling of the T-cell repertoire between baseline and surgery, and strong upregulation of cytotoxic CD8 + T-cell genes between baseline and samples taken early-on treatment. Of note, this trial was stopped early following an interim analysis which demonstrated more relapse events in the standard of care group. Further predictive probability modelling showed neoadjuvant plus adjuvant DAB + TRAM would be superior to standard of care, leading to closure of the standard of care group.

Long et al. reported the single-arm phase II ‘NeoCombi’ trial which evaluated pathological response after neoadjuvant DAB + TRAM for resectable stage III BRAF^V600^ mutant melanoma [27]. Thirty-five patients were enrolled, all of whom had a pathologic response. 17 (49%) had a pCR and 18 (51%) had a pathologic partial response (pPR). A total of 20 (57%) patients recurred; 14 with distant metastases, eight of whom had brain metastases. Median DMFS was 30.8 months in the overall population, 38.0 months in patients with a pCR, and 27.7 months in patients with a pPR. 2-yr OS was 93.8%; median OS was not reached. Neoadjuvant treatment was well tolerated with grade 3–4 AEs occurring in 29% of patients. In biomarker analysis, pCR was correlated with a higher proportion of Ki67-positive melanoma cells at baseline, CD8+ T-cell infiltration and melanoma PD-L1 expression.

In the ‘REDUCTOR’ trial, Blankenstein et al. evaluated the effect of short-term DAB + TRAM on the rate of conversion from unresectable to resectable disease for patients with locally advanced stage III or oligometastatic stage IV melanoma with BRAF^V600E/K^ mutation [29]. The trial is ongoing with 20 patients accrued. Two out of twenty patients progressed and could not have surgery. Sixteen out of eighteen underwent R0 resection and one out of eighteen underwent R1 resection. Pathologic response rates were pCR in 7 (35%), pPR in 7 (35%), no response in 3 (15%), and ‘not assessed’ in 3 (no surgical resection). 2-yr OS was 84%; median OS was not reached. Grade 3 AEs were observed in 3 (15%) patients.

### 3.2. Neoadjuvant Immunotherapy

Neoadjuvant checkpoint inhibitor immunotherapy has been studied in 3 phase II trials. Amaria et al. reported a randomized phase II trial with two arms: single-agent NIVO (3 mg/kg every 2 weeks for four cycles; arm A) and combination IPI plus NIVO (3 mg/kg IPI + 1 mg/kg NIVO every 3 weeks for three cycles; arm B) [20]. Notably, the trial closed early due to: (1) disease progression with synchronous metastasis and local progression preventing surgery in the NIVO group; (2) grade 3 AEs in 73% of patients on IPI + NIVO compared to 8% in the single-agent NIVO group. A total of 23 patients were recruited, with 12 patients in arm A and 11 in arm B. A pCR was obtained in 3 (25%) patients in arm A and 5 (45%) patients in arm B. Overall, IPI + NIVO showed improved PFS, RFS, DMFS and OS but none of these differences were statistically significant. When compared to non-responders, responders’ specimen analysis at baseline and early on-treatment had higher CD8+ T cell infiltrate and tumor cell PD-L1 expression, as well as more T cell clones.

In the ‘OpACIN-neo’ trial, Rozeman et al. randomized patients with resectable stage III melanoma with nodal metastases only to three regimens of combination IPI and NIVO. The primary outcomes were pCR and grade 3–4 AEs [30,31]. A total of 89 patients were randomized into three groups: (A) two cycles of IPI 3 mg/kg + NIVO 1 mg/kg every 3 weeks (30 patients); (B) two cycles of IPI 1 mg/kg + NIVO 3 mg/kg every 3 weeks (30 patients); or (C) two cycles of IPI 3 mg/kg every 3 weeks directly followed by two cycles of NIVO 3 mg/kg every two weeks (29 patients). pCR was seen in 14 (47%) patients in group A, 17 (57%) patients in group B, and 6 (23%) patients in group C. Up to 80% of patients in group A experienced any amount of pathological response (i.e., pCR , near pCR or pPR) as compared to 77% of patients in group B, and 65% of patients in group C. Grade 3–4 AEs occurred in 12 (40%) patients in group A, 6 (20%) patients in group B, and 13 (50%) patients in group C. After a median follow-up of 24.6 months, median RFS was not reached. Only one of the 64 patients with pCR had relapsed, as opposed to 65% of the non-responders. Estimated 2-year RFS was 84% for the total patient population, 97% for those with a pathologic response, and 36% for those without a pathologic response. The authors concluded that two cycles of neoadjuvant IPI 1 mg/kg + NIVO 3 mg/kg without adjuvant treatment lead to a durable RFS in more than 80% of patients with limited AEs [30]. Finally, an exploratory biomarker analysis of samples taken at baseline showed an associated between IFN-γ signature and relapse status, while PD-L1 expression on tumor cells had no association with pathologic response to treatment.

The OpACIN-neo trial provided justification for an extension cohort to tailor both the surgical approach and systemic therapy approach based on personalized tumor response after neoadjuvant IPI and NIVO in resectable stage III melanoma (PRADO trial) [32]. The primary outcome is whether therapeutic lymph node dissection (TLND) could be omitted in patients who achieved a major pathologic response (MPR) in an ‘index node’ marked prior to neoadjuvant therapy. MPR was defined as complete or near complete pathologic response (less than 10% of viable tumor cells). Additionally, the trial aims to establish if adjuvant therapy improves oncologic outcomes for non-responders. Initial results presented at ASCO2020 showed 99 patients currently enrolled, of whom 60 (61%) had an MPR. This response rate allowed omission of TLND in 58 (97%) of the patients with MPR, thereby reducing surgical morbidity. 28 patients did not respond to immunotherapy, with seven developing distant metastases before the index node could be resected. Adjuvant NIVO was given to eight patients and adjuvant DAB + TRAM to seven others. RFS data was not published due to data immaturity.

### 3.3. Neoadjuvant Intralesional Therapy

Neoadjuvant intralesional therapy for melanoma has been studied in 2 phase II trials using talimogene laherparepvec (T-VEC) and ‘CheckMate Pharmaceuticals’^©^, toll-like receptor 9 agonist (CMP-001). Andtbacka et al. reported results of a randomized controlled trial evaluating the effect of neoadjuvant intralesional T-VEC on pathologic response and survival outcomes in patients with resectable stage IIIB/C/IVA melanoma and at least 1 injectable cutaneous, subcutaneous or nodal lesion [33]. 150 patients were randomized to either 6 doses of intralesional T-VEC followed by surgery (arm 1; 76 patients) or upfront surgery (arm 2; 74 patients). Surgery occurred as planned in only 57 (75%) patients in arm 1 and 70 (93%) patients in arm 2. In arm 1, 11 (15%) patients progressed on treatment. In arm 2, 17 (23%) patients recurred within 14 weeks after surgery. For those who underwent surgery, the pCR rate in arm 1 was 21%. There was a higher rate of R0 resection in arm 1 (56.1%) compared to arm 2 (40.6%) [34]. Survival data published in 2019 showed improved 2-year RFS and OS in the neoadjuvant T-VEC group [35]. 2-year RFS was 29.5% in arm 1 versus 16.5% in arm 2 (HR 0.75, *p* < 0.07). Additional sensitivity analysis excluding non-R0 events showed statistically significant improvement in 2-year RFS in the neoadjuvant T-VEC arm (50.5% in arm 1 versus 30.2% in arm 2; HR 0.66, *p* < 0.038). 2-year OS rates were 88.9% in arm 1 compared to 77.4% in arm 2 (HR 0.49, *p* < 0.05) [35]. This highlights the importance of complete R0 resection surgery as an important factor in outcome. Intralesional T-VEC lead to a significant increase CD8+ cell density and PD-L1 in tumor specimens after treatment, which in turn correlated with longer RFS and OS.

Davar et al. undertook a single-arm phase II trial to evaluate neoadjuvant NIVO and intralesional CMP-001 in patients with high-risk resectable stage IIIB/C/D melanoma [36]. 20 patients were enrolled who received neoadjuvant subcutaneous CMP-001, followed by CMP-001 with concomitant NIVO over 7 weeks, followed by surgery, and then adjuvant NIVO and CMP-001 for a total of 48 weeks. Sixteen patients were evaluated for response, of which 76% had a MPR, with 10 (63%) pCRs and 2 (13%) pPRs. Biomarker analysis revealed an increase in CD8 T cell infiltrates and circulating PD1 +/Ki67+ CD8+ T cells in responders. The authors concluded that this regimen was not only safe, but also that using intralesional CMP-001 potentiates the efficacy of immune checkpoint blockade.

### 3.4. Quality of Studies

Quality assessments are summarized in Appendix B and Appendix C. Risk of bias in the four randomized controlled trials was assessed using the Revised Cochrane risk-of-bias tool for randomized trials (RoB 2). Journal articles, conference abstracts and trial registry records were used to help inform the risk-of-bias assessment. Overall, studies contained a low risk of bias with only some concerns. The quality of single-arm trials assessed using the modified Newcastle-Ottawa Scale was consistently high pertaining to selection of candidates and outcomes assessment.

## 4. Discussion

The multimodal management of stage III melanoma and oligometastatic stage IV melanoma is rapidly evolving. Due to the high-risk of recurrence and death despite aggressive surgical intervention, this population is an attractive target for novel treatment paradigms. While the effectiveness of neoadjuvant treatments has been well established in many solid organ malignancies (e.g., breast, rectal and gastric cancer), this review highlights noteworthy preliminary evidence of the exciting potential of contemporary neoadjuvant therapies in palpable/locally advanced Stage III and resectable stage IV melanoma. Based on the established rationale for neoadjuvant therapies in other cancers, the studies reviewed suggest that: (1) neoadjuvant therapy induces frequent and substantial pathologic response; (2) pCR may be a valuable prognostic indicator for RFS, or even OS after neoadjuvant therapy; (3) neoadjuvant therapy is safe, with reasonable treatment toxicities; (4) neoadjuvant therapy may convert well-selected unresectable melanoma to resectable disease; (5) overall oncologic outcomes (RFS, DMFS, OS) may be improved with a neoadjuvant approach.

Eight phase II trials were included in this review with a total of 450 patients. Three studies used neoadjuvant anti-BRAF/MEK targeted therapy, three used anti-PD-1/CTLA-4 immune checkpoint inhibition and two used intralesional therapy. While formal quantitative analysis was not feasible, outcomes were more homogeneous, so qualitative evaluation of pathologic response, recurrence and survival was completed. Because international consensus has grown in support of agreed standards in trial design (e.g., *International Neoadjuvant Melanoma Consortium* trial design guidelines) future results from these preliminary studies should be quantitatively comparable, with similar populations and endpoints [37]. In fact, a very recently published pooled analysis from the International Neoadjuvant Melanoma Consortium reported similar results to our systematic review [38].

Pathologic response varied across all trials. The use of targeted therapy yielded a pCR ranging from 35–58%. In trials using immunotherapy, pCR was achieved in 25–57%. Complete pathologic response (pCR) was most consistently observed in patients who received neoadjuvant combination IPI 1 mg/kg + NIVO 3 mg/kg, as described in arm B of the OpACIN-neo trial and the PRADO trial. Finally, in trials using intralesional therapy, pCR ranged from 21–76%.

Although the rates of pCR at surgical resection seem comparable between neoadjuvant targeted therapy and immunotherapy, recurrence-free survival in patients with pCR differs. When RFS was assessed based on response to treatment, data showed that patients with a complete or near-complete pathologic response had better RFS than non-responders across all trials. However, patients who received neoadjuvant targeted therapy had a higher risk of relapse than those treated with neoadjuvant immunotherapy. In the NeoCombi trial, although the number of patients in follow-up is small, almost half of the patients with pCR had recurred at the time of data cut-off. In the neoadjuvant immunotherapy trials, pCR is strongly associated with an absence of relapse. Remarkably, of the three neoadjuvant immunotherapy trials, only one of 105 patients with pCR relapsed. While more long-term survival data is needed to determine whether pCR correlates with OS, the reviewed studies suggest that pCR likely correlates with RFS.

The results of trials using intralesional therapy show that there is value in considering neoadjuvant intralesional treatment in patients with in-transit disease. When combined with systemic immunotherapy, CMP-001 increases immune activation both in the tumor and systemically. These results suggest that synergistically combining different therapy modalities offers increased treatment value for in-transit disease. Of note, AEs were minimal in intralesional trials.

The lowest rates of grade 3–4 AEs and highest pCR rates were seen with the DAB + TRAM and combination IPI + NIVO at the doses of IPI 1 mg/kg + NIVO 3 mg/kg neoadjuvant regimens. When directly compared, grade 3–4 AEs and rates of pCR are similar. However, neoadjuvant immunotherapy showed longer RFS. These results provide justification for trials assessing other treatment regimen combinations, such as the ‘NeoTrio’ trial, which evaluates targeted therapy plus anti-PD-1 immune checkpoint blockade [39]. Perhaps most interesting in terms of trial concept is the PRADO trial, which attempts to personalize care based on treatment response, and de-escalate the surgical management of node positive melanoma after neoadjuvant combination IPI + NIVO. Response to treatment was substantial with 60% of patients achieving a major pathologic response, thus avoiding TLND. Unfortunately, long-term follow up is required to determine local recurrence rates and survival in those who avoided TLND. If results are favorable, the PRADO trial will be practice changing—major surgery and the associated morbidity could be avoided for a significant subset of patients.

One area of concern regarding the neoadjuvant approach is losing the surgical ‘window of opportunity’ for non-responders. For that reason, careful patient selection of those likely to benefit from a neoadjuvant approach is crucial. In the trials reviewed, for T-VEC + surgery versus surgery alone, 25% of patients in the neoadjuvant T-VEC arm did not have surgery as planned versus 95% in the surgery alone arm. Notably, the trial by Amaria et al. comparing neoadjuvant NIVO versus IPI + NIVO was stopped early for safety, in part due to 17% of patients in the NIVO arm progressing on treatment and losing the opportunity for primary surgery. Interestingly, despite evidence of a lower response rate as compared to immunotherapy, no patient who was initially resectable at trial entry in the two targeted therapy trials involving this patient population progressed during neoadjuvant therapy. While it is certainly *not* established that primary surgical resection for non-responders provides a recurrence or survival benefit, surgery in this population may provide palliation and improved quality of life.

Predictive biomarker analysis of responders and non-responders may help determine which populations will benefit from neoadjuvant therapy. For example, Huang et al. discovered that in resectable stage III or IV melanoma, a single dose of anti-PD1 immunotherapy (pembrolizumab) resulted in T-cell reinvigoration at 7 days post-treatment [40]. This serum-detectable response was not only associated with pCR and pPR, but also DFS and OS at 2-years. Results of biomarker analysis in these neoadjuvant trials has thus far lead to several important hypotheses. Amaria et al. established that response to anti-PD-1 monotherapy may be dependent on a higher “tumor-educated T cell repertoire” [20]. Rozeman et al. suggest that IFN-gamma signature may be a good biomarker to assess outcome after neoadjuvant IPI and NIVO as it carries an association with relapse status, as also suggested by the adjuvant targeted therapy literature [31,41]. Finally, in the NeoCombi trial, patients who had a pCR had more Ki-67-positive melanoma cells in their biopsy specimens at baseline, consistent with signs of an immune response preceding treatment [27].

Whether neoadjuvant immunotherapy will replace adjuvant approaches in melanoma remains an open question. Ongoing trials, such as the SWOG1801 study, aim to answer this question [42]. The variability of response amongst preliminary neoadjuvant trials shows the importance of identifying strategies to predict the effectiveness of immuno-, targeted, and intralesional therapy. These methods will inform clinical decision making regarding upfront surgery versus neoadjuvant systemic therapy for resectable stage III and IV melanoma.

The strengths of this systematic review include a systematic approach to study inclusion, pragmatic reporting of study results, and maximizing data capture in the setting of sparse literature. The limitations of this review relate to the significant clinical heterogeneity between studies and the absence of large phase III studies. Consequently, making firm clinically relevant conclusions is not feasible. Although the inclusion of abstracts in a systematic review may affect the appraisal of heterogeneity, risk-of-bias assessment, and the reliability of reported results, this review included abstracts due to the scarcity of available evidence on this topic [43]. Finally, although quality appraisal revealed a low risk-of-bias amongst studies, this must be interpreted in the context of small sample sizes, impacting the generalizability of these trials’ results.

## 5. Conclusions

With the remarkable success of adjuvant immuno-, targeted, and intralesional therapies for melanoma, the rationale to evaluate these contemporary therapies in the neoadjuvant setting is clear. This review highlights the encouraging preliminary results of 8 phase II trials evaluating neoadjuvant therapies for high-risk palpable stage III and resectable stage IV melanoma. Results suggest that the neoadjuvant approach is not only safe and feasible, but also dramatically improves pathologic response and very likely RFS. In the future, understanding predictors of pCR and its correlation with long-term oncologic outcomes will be paramount. More mature data of the phase II trials and future phase III trials will help determine whether contemporary neoadjuvant therapy can supplant adjuvant therapy as the standard of care for resectable stage III and IV melanoma.

## Figures and Tables

**Table 1:** Summary of phase II randomized controlled and single-arm trials using neoadjuvant contemporary therapies for resectable stage III/IV melanoma.

Trial	Design	Patient Population	Regimen	N	Grade 3–4 AEs (% pts)	pCR (%)	Median RFS (95% CI) (mo)	Med FU (IQR) (mo)
NCT02231775 Amaria et al. Lancet Oncol 2018	Randomized, open-label, phase II	Stage IIIB-D, resectable IV BRAF V600E/K	dab + tram × 8 w → Surgery → dab + tram × 44 w	14	15	50	19.7 (16.2-NR) HR 0.016 (*p* < 0.0001)	18.6 (14.6–23.1)
			Upfront surgery + consideration of adjuvant therapy	7	NA	NA	2.9 (1.7-NR)	
NCT01972347 NeoCombi Long et al. Lancet Oncol 2019	Single-arm, open-label, phase II	Clinical Stage IIIB-C BRAF V600E/K	dab + tram × 12 w → Surgery → dab + tram × 40 w	35	29	49	23.3 (17.7-NR)	27 (21–36)
NTR4654 REDUCTOR trial Blankenstein et al. J Clin Oncol 2019	Single-arm, phase II	Locally advanced Stage III, oligometastatic IV BRAF V600E/K	dab + tram × 8 w → Surgery	20	15	35	9	28
NCT02519322 Amaria et al. Nature 2018	Randomized, phase II	Clinical stage IIIB-D, oligometastatic IV	nivo3 q2w × 4	12	8	25	NR	15
			ipi3 + nivo1 q3w × 3	11	73	45	NR	15.6
NCT02977052 OpACIN-neo Rozeman et al. Lancet Oncol 2019 Rozeman et al. ASCO 2020	Randomized, open-label, phase II	Resectable stage IIIB-D (nodal metastases only)	ipi3 + nivo1 q3w × 2	30	40	47	NR	24.6
			ipi1 + nivo3 q3w × 2	30	20	57		
			ipi3, 3w later: ipi3 + nivo3, 2w later: nivo3	26	50	23		
NCT02977052 PRADO Blank et al. ASCO 2020	Extension cohort of OpACIN-neo trial	Resectable stage IIIB-D (nodal metastases only)	Marker in ILN → ipi1 + nivo3 q3w × 2 → ILN resection → no further therapy if MPR vs. TLND ± adjuvant nivo or TT × 52w	99	24	61	NR	NR
NCT02211131 Andtbacka et al. J Clin Oncol 2018 Dummer et al. J Clin Oncol 2019 Dummer et al. Annals Onc 2019	Randomized, open label, phase II	Resectable stage IIIB-D/IV1a and ≥ 1 injectable cutaneous, subcutaneous or nodal lesion	6 × intralesional T-VEC → Surgery	76	NA	21	NR	31.2
			Surgery alone	74		NA		
NCT03618641 Davar et al. J Imm Cancer 2019	Single-arm, phase II	Stage IIIB-D	Subcutaneous CMP-001 × 1, then intralesional CMP-001 × 7 + nivo 240 mg q2w × 3 → Surgery → nivo 480 mg q4w + s/c CMP-001 q4w × 48w	16	NA	76	NA	NA

AEs, adverse events. pCR, pathologic complete response. RFS, relapse-free survival. NR, not reached. FU, follow-up. HR, hazard ratio. NA, not assessed. ILN, index lymph node. MPR, major pathologic response. TLND, therapeutic lymph node dissection. TT, targeted therapy.

## Appendix A. Search Algorithm for Embase, Ovid MEDLINE and Cochrane Central Register of Controlled Trials

Database: Embase Classic + Embase <1947 to 2020 February 13>, Ovid MEDLINE(R) ALL <1946 to 13 February 2020>, EBM Reviews—Cochrane Central Register of Controlled Trials <January 2020>

Search Strategy:

--------------------------------------------------------------------------------

1. Melanoma/(222,910)
2. Melanoma.tw,kw. (273,562)
3. 1 or 2 (326,579)
4. Neoadjuvant *.mp. (108,881)
5. neo adjuvant *.tw,kw. (10,747)
6. 4 or 5 (115,582)
7. 3 and 6 (1161)
8. Preoperative Care/or Perioperative Care/(166,273)
9. (pre-operat * or perioperat *).tw,kw. (346,048)
10. (presurg * or before surg * or pre surg * or preoperat *).tw,kw. (986,531)
11. 8 or 9 or 10 (1,293,935)
12. 3 and 11 (5566)
13. Immunotherapy/(131,805)
14. checkpoint blockade.mp. (9325)
15. (ipilimumab or nivolumab or pembrolizumab).mp. (39,087)
16. (immunotherap* or immune therap*).tw,kw. (235,711)
17. Vemurafenib.mp. (9877)
18. dabrafenib.mp. (5397)
19. encorafenib.mp. (576)
20. Proto-Oncogene Proteins B-raf/ai [Antagonists & Inhibitors] (1663)
21. trametinib.mp. (6157)
22. cobimetinib.mp. (499)
23. binimetinib.mp. (910)
24. Protein Kinase Inhibitors/(55,010)
25. MEK inhibitor *.tw,kw. (13,721)
26. braf inhibitor *.tw,kw. (5901)
27. targeted therap *.tw,kw. (125,246)
28. t vec.mp. (482)
29. Imlygic.mp. (135)
30. talimogene laherparepvec.mp. (1103)
31. or/13-30 (492,162)
32. 12 and 31 (518)
33. 7 or 32 (1554)
34. limit 33 to english language (1331)
35. 34 use medall (354) Medline
36. 34 use cctr (189) Cochrane
37. exp melanoma/(259,167)
38. melanoma.tw. (269,019)
39. 37 or 38 (331,962)
40. exp neoadjuvant therapy/(40,087)
41. (neoadjuvant * or neo adjuvant *).tw. (104,605)
42. 40 or 41 (113,911)
43. 39 and 42 (1157)
44. preoperative care/or exp preoperative treatment/or perioperative period/(175,695)
45. (presurg * or before surg * or pre surg * or preoperat * or pre operat * or perioperat*).tw. (1,228,695)
46. 44 or 45 (1,291,802)
47. 39 and 46 (5600)
48. (ipilimumab or nivolumab or pembrolizumab).mp. (39,087)
49. (checkpoint blockade or checkpoint inhibitor *).tw. (31,158)
50. cancer immunotherapy/(59,391)
51. (immunotherapy * or immune therap *).tw. (218,969)
52. Vemurafenib.tw. (5724)
53. dabrafenib.tw. (2888)
54. encorafenib.tw. (275)
55. exp B Raf kinase inhibitor/(10,372)
56. exp mitogen activated protein kinase inhibitor/(31,011)
57. MEK inhibitor *.tw. (13,472)
58. braf inhibitor *.tw. (5633)
59. target * therap *.tw. (129,800)
60. talimogene laherparepvec/(796)
61. t vec.tw. (450)
62. talimogene laherparepvec.tw. (585)
63. Imlygic.tw. (130)
64. programmed death 1 receptor/(19,416)
65. or/48-64 (443,289)
66. 47 and 65 (505)
67. 43 or 66 (1535)
68. limit 67 to english language (1,318)
69. 68 use emczd (800) Embase
70. 35 or 36 or 69 (1343)
71. remove duplicates from 70 (1013)
72. 71 use medall (354)
73. 71 use emczd (507)
74. 71 use cctr (152)

## Appendix B

**Table A1:** Risk of bias summary for the randomized controlled trials using the cochrane risk of bias tool.

Studies with Intention-to-Treat		Outcome	Randomization Process	Deviations from Intended Interventions	Missing Outcome Data	Measurement of the Outcome	Selection of the Reported Result	Overall		
	NCT02231775 [28]	pCR								Low risk
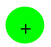
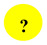
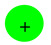
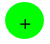
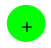
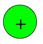
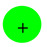
	NCT01972347 [20]	pCR								Some concerns
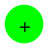
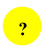
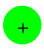
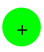
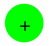
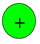
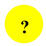
	NCT02977052 [31,30]	pCR								High risk
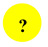
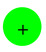
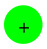
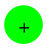
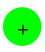
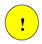
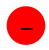
	NCT02211131 [33,34,35]	pCR								
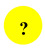
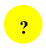
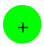
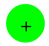
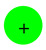
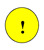

## Appendix C

**Table A2:** Newcastle-Ottawa quality assessment scale for single-arm trials.

Trial	Selection			Outcome			Total Stars (/6)
	(1)	(3)	(4)	(1)	(2)	(3)	
NCT01972347 [27]	∗	∗	∗	∗	∗	∗	6
NTR4654 [29]	∗	∗	∗	∗	∗	∗	6
NCT02977052 [32]	∗	∗	∗	∗	∗	∗	6
NCT02211131 [36]	∗	∗	∗	∗	∗	∗	6
